# Supplementary material for: Persistence of Colistin Resistance and mcr-1.1-Positive E. coli in Poultry Despite Colistin Ban in Japan
Source: Antibiotics (Basel). 2025 Apr 1;14(4):360. doi: 10.3390/antibiotics14040360 (PMC12024320; doi:10.3390/antibiotics14040360)
Supplement: Supplementary file 1 [file antibiotics-14-00360-s001.zip › Figure-S1.pdf]

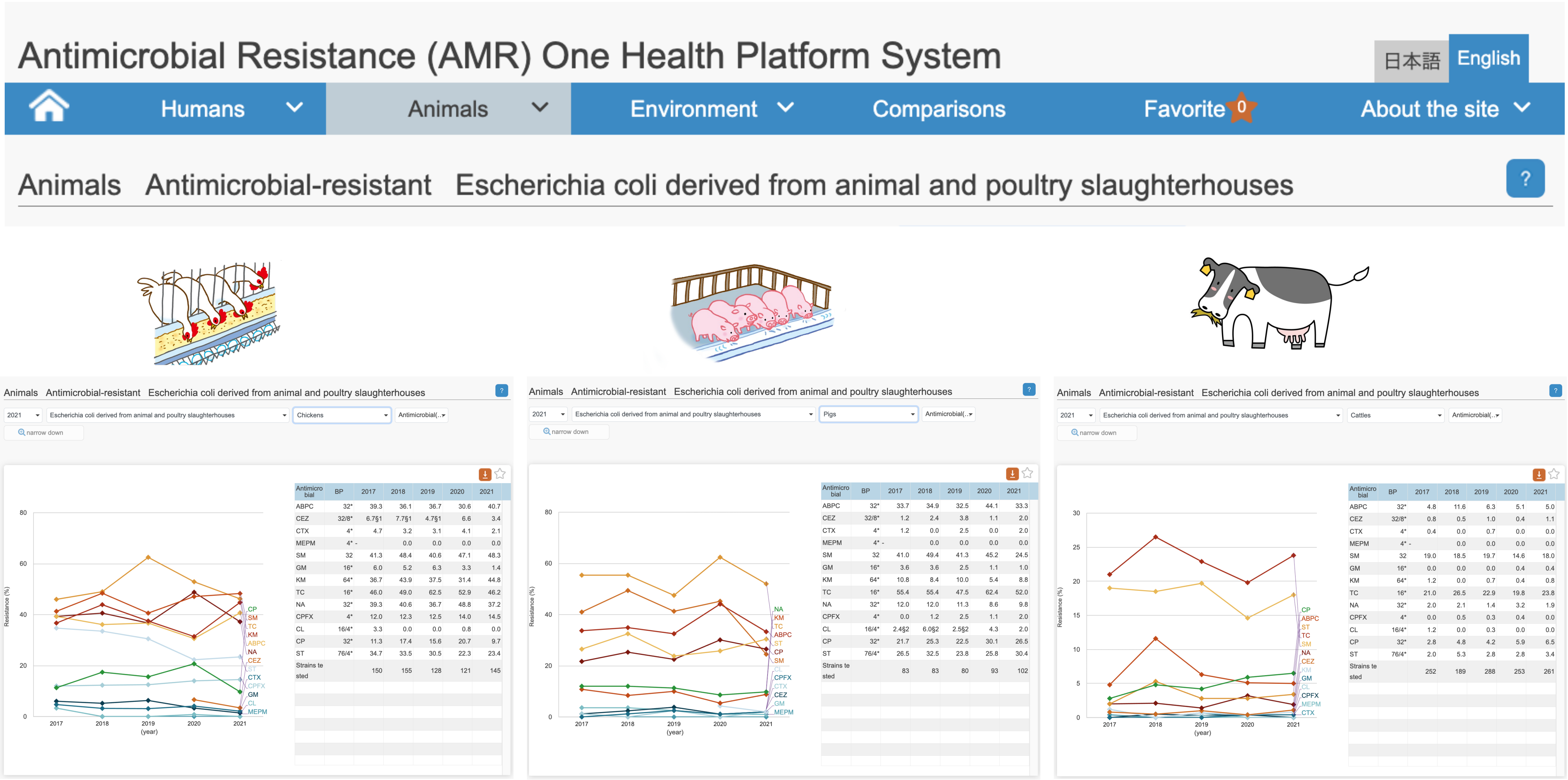

Chickens

Antimicrobial	BP	2017	2018	2019	2020	2021
ABPC	32*	39.3	36.1	36.7	30.6	40.7
CEZ	32/8*	6.7§1	7.7§1	4.7§1	6.6	3.4
CTX	4*	4.7	3.2	3.1	4.1	2.1
MEPM	4* -		0.0	0.0	0.0	0.0
SM	32	41.3	48.4	40.6	47.1	48.3
GM	16*	6.0	5.2	6.3	3.3	1.4
KM	64*	36.7	43.9	37.5	31.4	44.8
TC	16*	46.0	49.0	62.5	52.9	46.2
NA	32*	39.3	40.6	36.7	48.8	37.2
CPFX	4*	12.0	12.3	12.5	14.0	14.5
CL	16/4*	3.3	0.0	0.0	0.8	0.0
CP	32*	11.3	17.4	15.6	20.7	9.7
ST	76/4*	34.7	33.5	30.5	22.3	23.4
Strains tested		150	155	128	121	145

Pigs

Antimicrobial	BP	2017	2018	2019	2020	2021
ABPC	32*	33.7	34.9	32.5	44.1	33.3
CEZ	32/8*	1.2	2.4	3.8	1.1	2.0
CTX	4*	1.2	0.0	2.5	0.0	2.0
MEPM	4* -		0.0	0.0	0.0	0.0
SM	32	41.0	49.4	41.3	45.2	24.5
GM	16*	3.6	3.6	2.5	1.1	1.0
KM	64*	10.8	8.4	10.0	5.4	8.8
TC	16*	55.4	55.4	47.5	62.4	52.0
NA	32*	12.0	12.0	11.3	8.6	9.8
CPFX	4*	0.0	1.2	2.5	1.1	2.0
CL	16/4*	2.4§2	6.0§2	2.5§2	4.3	2.0
CP	32*	21.7	25.3	22.5	30.1	26.5
ST	76/4*	26.5	32.5	23.8	25.8	30.4
Strains tested		83	83	80	93	102

Cattles

Antimicrobial	BP	2017	2018	2019	2020	2021
ABPC	32*	4.8	11.6	6.3	5.1	5.0
CEZ	32/8*	0.8	0.5	1.0	0.4	1.1
CTX	4*	0.4	0.0	0.7	0.0	0.0
MEPM	4* -		0.0	0.0	0.0	0.0
SM	32	19.0	18.5	19.7	14.6	18.0
GM	16*	0.0	0.0	0.0	0.4	0.4
KM	64*	1.2	0.0	0.7	0.4	0.8
TC	16*	21.0	26.5	22.9	19.8	23.8
NA	32*	2.0	2.1	1.4	3.2	1.9
CPFX	4*	0.0	0.5	0.3	0.4	0.0
CL	16/4*	1.2	0.0	0.3	0.0	0.0
CP	32*	2.8	4.8	4.2	5.9	6.5
ST	76/4*	2.0	5.3	2.8	2.8	3.4
Strains tested		252	189	288	253	261

Figure S1: AMR One Health Platform System for Animals, Antimicrobial resistant *E. coli*.
https://amr-onehealth-platform.ncgm.go.jp/resistantBacteria/201
