# Supplementary material for: Persistence of Colistin Resistance and mcr-1.1-Positive E. coli in Poultry Despite Colistin Ban in Japan
Source: Antibiotics (Basel). 2025 Apr 1;14(4):360. doi: 10.3390/antibiotics14040360 (PMC12024320; doi:10.3390/antibiotics14040360)
Supplement: Supplementary file 1 [file antibiotics-14-00360-s001.zip › Table-S1.pdf]

Supplementary Table S1. Antimicrobial susceptibility profiles for 147 *E. coli* strains in this study.

Strain	Farm	Cluster group	PIPC	TAZ/PIP	CFPM	CAZ	CZOP	CL	FOM	IPM	MEPM	GM	AMK	TOB	MINO	LVFX	CPFX	DRPM	AZT	TMP/SMX
363	E	1	≥128	<4/2	<0.5	<1	<0.5	<1	<32	<0.5	<0.5	≥16	<4	4	2	2	1	<1	<2	≥4/76
339	D	1	≥128	<4/2	<0.5	<1	<0.5	<1	<32	<0.5	<0.5	≥16	<4	4	8	2	1	<1	<2	≥4/76
252-4	E	1	≥128	<4/2	<0.5	<1	<0.5	<1	<32	<0.5	<0.5	≥16	<4	4	<1	<0.5	<0.25	<1	<2	<0.5/9.5
254-2	E	1	<2	<4/2	<0.5	<1	<0.5	<1	<32	<0.5	<0.5	≥16	<4	4	<1	<0.5	<0.25	<1	<2	≥4/76
287-1	B	1	<2	<4/2	<0.5	<1	<0.5	<1	<32	<0.5	<0.5	≥16	<4	4	<1	<0.5	<0.25	<1	<2	≥4/76
133	A	1	<2	<4/2	<0.5	<1	<0.5	<1	<32	<0.5	<0.5	≥16	<4	4	<1	<0.5	<0.25	<1	<2	≥4/76
121	A	1	<2	<4/2	<0.5	<1	<0.5	<1	<32	<0.5	<0.5	≥16	<4	4	<1	<0.5	<0.25	<1	<2	≥4/76
381	E	1	<2	<4/2	<0.5	<1	<0.5	<1	<32	<0.5	<0.5	≥16	<4	4	<1	<0.5	<0.25	<1	<2	≥4/76
259-2-A	E	1	<2	<4/2	<0.5	<1	<0.5	<1	<32	<0.5	<0.5	≥16	<4	4	<1	<0.5	<0.25	<1	<2	≥4/76
251	E	1	<2	<4/2	<0.5	<1	<0.5	<1	<32	<0.5	<0.5	≥16	<4	4	<1	<0.5	<0.25	<1	<2	<0.5/9.5
4-2	B	1	<2	<4/2	<0.5	<1	<0.5	<1	<32	<0.5	<0.5	≥16	<4	4	<1	<0.5	<0.25	<1	<2	<0.5/9.5
371	E	1	<2	<4/2	<0.5	<1	<0.5	<1	<32	<0.5	<0.5	≥16	<4	4	<1	<0.5	<0.25	<1	<2	<0.5/9.5
260-A	E	1	<2	<4/2	<0.5	<1	<0.5	<1	<32	<0.5	<0.5	≥16	<4	4	<1	<0.5	<0.25	<1	<2	<0.5/9.5
255-2	E	1	<2	<4/2	<0.5	<1	<0.5	<1	<32	<0.5	<0.5	≥16	<4	4	<1	<0.5	<0.25	<1	<2	<0.5/9.5
15	B	1	<2	<4/2	<0.5	<1	<0.5	<1	<32	<0.5	<0.5	8	<4	<1	<1	<0.5	<0.25	<1	<2	≥4/76
253-2	E	1	≥128	<4/2	<0.5	<1	<0.5	<1	<32	<0.5	<0.5	8	<4	2	<1	2	1	<1	<2	≥4/76
183-2	B	2	<2	<4/2	<0.5	<1	<0.5	<1	<32	<0.5	<0.5	<1	<4	<1	<1	<0.5	<0.25	<1	<2	<0.5/9.5
183-1	B	2	<2	<4/2	<0.5	<1	<0.5	<1	<32	<0.5	<0.5	<1	<4	<1	<1	<0.5	<0.25	<1	<2	<0.5/9.5
180	B	2	<2	<4/2	<0.5	<1	<0.5	<1	<32	<0.5	<0.5	<1	<4	<1	<1	<0.5	<0.25	<1	<2	<0.5/9.5
179	B	2	<2	<4/2	<0.5	<1	<0.5	<1	<32	<0.5	<0.5	<1	<4	<1	<1	<0.5	<0.25	<1	<2	<0.5/9.5
177	B	2	<2	<4/2	<0.5	<1	<0.5	<1	<32	<0.5	<0.5	<1	<4	<1	<1	<0.5	<0.25	<1	≥32	<0.5/9.5
175	B	2	<2	<4/2	<0.5	<1	<0.5	<1	<32	<0.5	<0.5	<1	<4	<1	<1	<0.5	<0.25	<1	<2	<0.5/9.5
172	B	2	<2	<4/2	<0.5	<1	<0.5	<1	<32	<0.5	<0.5	<1	<4	<1	<1	<0.5	<0.25	<1	4	<0.5/9.5
171-2	B	2	<2	<4/2	<0.5	<1	<0.5	<1	<32	<0.5	<0.5	<1	<4	<1	<1	<0.5	<0.25	<1	<2	<0.5/9.5
216	D	2	<2	<4/2	<0.5	<1	<0.5	<1	<32	<0.5	<0.5	<1	<4	<1	<1	<0.5	<0.25	<1	<2	<0.5/9.5
207	D	2	<2	<4/2	<0.5	<1	<0.5	<1	<32	<0.5	<0.5	<1	<4	<1	<1	<0.5	<0.25	<1	<2	<0.5/9.5
53-1-2	C	2	<2	<4/2	<0.5	<1	<0.5	<1	<32	<0.5	<0.5	<1	<4	<1	2	<0.5	<0.25	<1	<2	<0.5/9.5
301-3	B	2	<2	<4/2	<0.5	<1	<0.5	<1	<32	<0.5	<0.5	<1	<4	<1	4	<0.5	<0.25	<1	<2	1/19
289	B	2	<2	<4/2	<0.5	2	<0.5	<1	<32	<0.5	<0.5	<1	<4	<1	<1	<0.5	<0.25	<1	<2	<0.5/9.5
288-1	B	2	<2	<4/2	<0.5	<1	<0.5	<1	<32	<0.5	<0.5	<1	<4	<1	<1	<0.5	<0.25	<1	<2	<0.5/9.5
287-2-G	B	2	<2	<4/2	<0.5	<1	<0.5	<1	<32	<0.5	<0.5	<1	<4	<1	2	<0.5	<0.25	<1	<2	<0.5/9.5
286-1	B	2	<2	<4/2	<0.5	<1	<0.5	<1	<32	<0.5	<0.5	<1	<4	2	<1	<0.5	<0.25	<1	<2	<0.5/9.5
284-2	B	2	<2	4/<4	<0.5	<1	<0.5	<1	<32	<0.5	<0.5	<1	<4	<1	2	<0.5	<0.25	<1	<2	<0.5/9.5
284-1	B	2	4	<4/2	<0.5	<1	<0.5	<1	<32	<0.5	<0.5	<1	<4	<1	<1	<0.5	<0.25	<1	<2	<0.5/9.5
283-1	B	2	<2	<4/2	<0.5	<1	<0.5	<1	<32	<0.5	<0.5	<1	<4	<1	<1	<0.5	<0.25	<1	<2	<0.5/9.5
281	B	2	<2	<4/2	<0.5	<1	<0.5	<1	<32	<0.5	<0.5	<1	<4	<1	<1	<0.5	<0.25	<1	<2	<0.5/9.5
164-1	B	2	<2	<4/2	<0.5	<1	<0.5	<1	<32	<0.5	<0.5	<1	<4	<1	<1	<0.5	<0.25	<1	<2	<0.5/9.5
4-1	B	2	<2	<4/2	<0.5	<1	<0.5	<1	<32	<0.5	<0.5	<1	<4	<1	<1	<0.5	<0.25	<1	<2	<0.5/9.5
257-2	E	2	<2	<4/2	<0.5	<1	<0.5	<1	<32	<0.5	<0.5	<1	<4	<1	<1	<0.5	<0.25	<1	<2	<0.5/9.5
171-1	B	2	<2	<4/2	<0.5	<1	<0.5	<1	<32	<0.5	<0.5	<1	<4	<1	<1	<0.5	<0.25	<1	<2	<0.5/9.5
169	B	2	<2	<4/2	<0.5	<1	<0.5	<1	<32	<0.5	<0.5	<1	<4	<1	<1	<0.5	<0.25	<1	<2	<0.5/9.5
252-1	E	2	<2	<4/2	<0.5	<1	<0.5	<1	<32	<0.5	<0.5	<1	<4	<1	<1	<0.5	<0.25	<1	<2	<0.5/9.5
217	D	2	<2	<4/2	<0.5	<1	<0.5	<1	<32	<0.5	<0.5	<1	<4	<1	<1	<0.5	<0.25	<1	<2	<0.5/9.5
301-2	B	2	<2	<4/2	<0.5	<1	<0.5	<1	<32	<0.5	<0.5	<1	<4	<1	<1	<0.5	<0.25	<1	<2	<0.5/9.5
301-1	B	2	<2	<4/2	<0.5	<1	<0.5	<1	<32	<0.5	<0.5	<1	<4	<1	2	<0.5	<0.25	<1	<2	<0.5/9.5
300-3	B	2	<2	<4/2	<0.5	<1	<0.5	<1	<32	<0.5	<0.5	<1	<4	<1	<1	<0.5	<0.25	<1	<2	<0.5/9.5
300-1	B	2	<2	<4/2	<0.5	<1	<0.5	<1	<32	<0.5	<0.5	<1	<4	<1	<1	<0.5	<0.25	<1	<2	<0.5/9.5
298-3	B	2	4	<4/2	<0.5	<1	<0.5	<1	<32	<0.5	<0.5	<1	<4	<1	<1	<0.5	<0.25	<1	<2	<0.5/9.5
298-2	B	2	<2	<4/2	<0.5	<1	<0.5	<1	<32	<0.5	<0.5	<1	<4	<1	<1	<0.5	<0.25	<1	<2	<0.5/9.5
298-1	B	2	<2	<4/2	<0.5	<1	<0.5	<1	<32	<0.5	<0.5	<1	<4	<1	<1	<0.5	<0.25	<1	<2	<0.5/9.5
297	B	2	<2	<4/2	<0.5	<1	<0.5	<1	<32	<0.5	<0.5	<1	<4	<1	<1	<0.5	<0.25	<1	<2	<0.5/9.5
295-2	B	2	<2	<4/2	<0.5	<1	<0.5	<1	<32	<0.5	<0.5	<1	<4	<1	2	<0.5	<0.25	<1	4	<0.5/9.5
295-1	B	2	<2	<4/2	<0.5	<1	<0.5	<1	<32	<0.5	<0.5	<1	<4	<1	<1	<0.5	<0.25	<1	<2	<0.5/9.5
294	B	2	<2	<4/2	<0.5	<1	<0.5	<1	<32	<0.5	<0.5	<1	<4	<1	<1	<0.5	<0.25	<1	<2	<0.5/9.5
293	B	2	<2	<4/2	<0.5	<1	<0.5	<1	<32	<0.5	<0.5	<1	<4	<1	<1	<0.5	<0.25	<1	<2	<0.5/9.5
292-1	B	2	<2	<4/2	<0.5	<1	<0.5	<1	<32	<0.5	<0.5	<1	<4	<1	<1	<0.5	<0.25	<1	<2	<0.5/9.5
291	B	2	<2	<4/2	<0.5	<1	<0.5	<1	<32	<0.5	<0.5	<1	<4	<1	<1	<0.5	<0.25	<1	<2	<0.5/9.5
290-A	B	2	<2	<4/2	<0.5	<1	<0.5	<1	<32	<0.5	<0.5	<1	<4	<1	<1	<0.5	<0.25	<1	<2	<0.5/9.5
290-1	B	2	<2	<4/2	<0.5	<1	<0.5	<1	<32	<0.5	<0.5	<1	<4	<1	<1	<0.5	<0.25	<1	<2	<0.5/9.5
90-2	D	2	16	4/8	<0.5	2	<0.5	<1	<32	<0.5	<0.5	<1	<4	<1	2	<0.5	<0.25	<1	16	<0.5/9.5
255-1	E	2	<2	<4/2	<0.5	<1	<0.5	<1	≥256	<0.5	<0.5	<1	<4	<1	<1	<0.5	<0.25	<1	<2	<0.5/9.5
257-1	E	2	<2	<4/2	<0.5	<1	<0.5	≥8	<32	<0.5	<0.5	<1	<4	<1	<1	<0.5	<0.25	<1	<2	<0.5/9.5
165	B	2	<2	<4/2	<0.5	<1	<0.5	≥8	64	<0.5	<0.5	<1	<4	<1	2	<0.5	<0.25	<1	<2	<0.5/9.5
365	E	3	<2	<4/2	<0.5	<1	<0.5	<1	<32	<0.5	<0.5	≥16	<4	2	<1	<0.5	<0.25	<1	<2	<0.5/9.5
362	E	3	<2	<4/2	<0.5	<1	<0.5	<1	<32	<0.5	<0.5	≥16	<4	2	<1	<0.5	<0.25	<1	<2	<0.5/9.5
361	E	3	<2	<4/2	<0.5	<1	<0.5	<1	<32	<0.5	<0.5	≥16	<4	2	<1	<0.5	<0.25	<1	<2	<0.5/9.5

**246-2**	E	3	<2	<4/2	<0.5	<1	<0.5	<1	<32	<0.5	<0.5	≥16	<4	2	<1	<0.5	<0.25	<1	<2	<0.5/9.5
**286-A**	B	3	<2	<4/2	<0.5	<1	<0.5	<1	<32	<0.5	<0.5	8	<4	<1	<1	<0.5	<0.25	<1	<2	<0.5/9.5
**17-3**	B	3	<2	<4/2	<0.5	<1	<0.5	<1	<32	<0.5	<0.5	≥16	<4	2	<1	<0.5	<0.25	<1	<2	<0.5/9.5
**383**	E	3	<2	<4/2	<0.5	<1	<0.5	<1	<32	<0.5	<0.5	≥16	<4	2	<1	<0.5	<0.25	<1	<2	<0.5/9.5
**335**	D	3	<2	<4/2	<0.5	<1	<0.5	<1	<32	<0.5	<0.5	≥16	<4	2	<1	<0.5	<0.25	<1	<2	<0.5/9.5
**288-A**	B	3	<2	<4/2	<0.5	<1	<0.5	<1	<32	<0.5	<0.5	≥16	<4	2	<1	<0.5	<0.25	<1	<2	<0.5/9.5
**372**	E	3	<2	<4/2	<0.5	<1	<0.5	<1	<32	<0.5	<0.5	≥16	<4	2	<1	<0.5	<0.25	<1	<2	<0.5/9.5
**369**	E	3	<2	<4/2	<0.5	<1	<0.5	<1	<32	<0.5	<0.5	≥16	<4	2	<1	<0.5	<0.25	<1	<2	<0.5/9.5
**368**	E	3	<2	<4/2	<0.5	<1	<0.5	<1	<32	<0.5	<0.5	≥16	<4	2	<1	<0.5	<0.25	<1	<2	<0.5/9.5
**367**	E	3	<2	<4/2	<0.5	<1	<0.5	<1	<32	<0.5	<0.5	≥16	<4	2	<1	<0.5	<0.25	<1	<2	<0.5/9.5
**412**	C	3	<2	<4/2	<0.5	<1	<0.5	<1	<32	<0.5	<0.5	8	<4	≥16	8	<0.5	<0.25	<1	<2	<0.5/9.5
**411**	C	3	<2	<4/2	<0.5	<1	<0.5	<1	<32	<0.5	<0.5	8	<4	≥16	8	<0.5	<0.25	<1	<2	<0.5/9.5
**48**	C	3	<2	<4/2	<0.5	<1	<0.5	<1	<32	<0.5	<0.5	8	<4	≥16	8	<0.5	<0.25	<1	<2	<0.5/9.5
**47-1**	C	3	8	<4/2	<0.5	<1	<0.5	<1	<32	<0.5	<0.5	≥16	<4	≥16	8	<0.5	<0.25	<1	16	<0.5/9.5
**364**	E	3	<2	<4/2	<0.5	<1	<0.5	<1	<32	<0.5	<0.5	8	<4	≥16	≥16	<0.5	<0.25	<1	<2	<0.5/9.5
**10-1**	B	3	≥128	<4/2	<0.5	<1	<0.5	<1	<32	<0.5	<0.5	2	≥64	16	<1	<0.5	<0.25	<1	<2	<0.5/9.5
**327**	D	4	≥128	<4/2	<0.5	4	<0.5	2	<32	<0.5	<0.5	<1	<4	<1	<1	<0.5	<0.25	<1	<2	≥4/76
**326**	D	4	32	<4/2	<0.5	4	<0.5	2	<32	<0.5	<0.5	<1	<4	<1	<1	<0.5	<0.25	<1	<2	≥4/76
**325**	D	4	64	<4/2	<0.5	4	<0.5	2	<32	<0.5	<0.5	<1	<4	<1	<1	<0.5	<0.25	<1	<2	≥4/76
**324**	D	4	64	<4/2	<0.5	4	<0.5	2	<32	<0.5	<0.5	<1	<4	<1	<1	<0.5	<0.25	<1	<2	≥4/76
**322**	D	4	64	<4/2	<0.5	4	<0.5	2	<32	<0.5	<0.5	<1	<4	<1	<1	<0.5	<0.25	<1	<2	≥4/76
**58-1**	C	4	≥128	<4/2	<0.5	4	<0.5	2	<32	<0.5	<0.5	<1	<4	<1	<1	<0.5	<0.25	<1	<2	≥4/76
**57**	C	4	≥128	<4/2	<0.5	4	<0.5	2	<32	<0.5	<0.5	<1	<4	<1	<1	<0.5	<0.25	<1	<2	≥4/76
**51**	C	4	≥128	<4/2	<0.5	4	<0.5	2	<32	<0.5	<0.5	<1	<4	<1	2	<0.5	<0.25	<1	<2	≥4/76
**137**	A	4	≥128	<4/2	<0.5	4	<0.5	2	<32	<0.5	<0.5	<1	<4	<1	<1	<0.5	<0.25	<1	<2	≥4/76
**134**	A	4	64	<4/2	<0.5	4	<0.5	2	<32	<0.5	<0.5	<1	<4	<1	<1	<0.5	<0.25	<1	<2	≥4/76
**125**	A	4	64	<4/2	<0.5	4	<0.5	2	<32	<0.5	<0.5	<1	<4	<1	<1	<0.5	<0.25	<1	<2	≥4/76
**123**	A	4	64	<4/2	<0.5	4	<0.5	2	<32	<0.5	<0.5	<1	<4	<1	<1	<0.5	<0.25	<1	<2	≥4/76
**250**	E	4	64	<4/2	<0.5	4	<0.5	2	<32	<0.5	<0.5	<1	<4	<1	<1	<0.5	<0.25	<1	<2	≥4/76
**328**	D	4	32	<4/2	<0.5	2	<0.5	2	<32	<0.5	<0.5	<1	<4	<1	<1	<0.5	<0.25	<1	<2	≥4/76
**45**	C	4	≥128	<4/2	<0.5	4	<0.5	2	<32	<0.5	<0.5	<1	<4	<1	<1	<0.5	<0.25	<1	<2	≥4/76
**44-1**	C	4	64	<4/2	<0.5	4	<0.5	2	<32	<0.5	<0.5	<1	<4	<1	<1	<0.5	<0.25	<1	<2	≥4/76
**43**	C	4	32	<4/2	<0.5	4	<0.5	2	<32	<0.5	<0.5	<1	<4	<1	<1	<0.5	0.5	<1	<2	≥4/76
**17-1**	B	4	≥128	<4/2	<0.5	4	<0.5	2	<32	<0.5	<0.5	<1	<4	2	<1	<0.5	<0.25	<1	<2	≥4/76
**252-2**	E	4	4	<4/2	<0.5	4	<0.5	2	<32	<0.5	<0.5	<1	<4	<1	<1	<0.5	<0.25	<1	<2	≥4/76
**136**	A	4	8	<4/2	<0.5	4	<0.5	2	<32	<0.5	<0.5	<1	<4	<1	<1	<0.5	<0.25	<1	<2	≥4/76
**254-1**	E	4	4	<4/2	<0.5	4	<0.5	2	<32	<0.5	2	<1	<4	2	<1	<0.5	<0.25	<1	<2	≥4/76
**135**	A	4	≥128	<4/2	<0.5	4	<0.5	4	<32	<0.5	<0.5	<1	<4	<1	<1	<0.5	<0.25	<1	<2	≥4/76
**130**	A	4	64	<4/2	<0.5	4	<0.5	4	<32	<0.5	<0.5	<1	<4	<1	<1	<0.5	<0.25	<1	<2	≥4/76
**258**	E	4	≥128	<4/2	<0.5	4	<0.5	4	<32	<0.5	<0.5	<1	<4	<1	<1	<0.5	<0.25	<1	<2	≥4/76
**42**	C	4	64	<4/2	<0.5	4	<0.5	4	<32	<0.5	<0.5	<1	<4	<1	<1	<0.5	<0.25	<1	<2	≥4/76
**17-2**	B	4	≥128	<4/2	<0.5	4	<0.5	4	<32	<0.5	<0.5	<1	<4	<1	<1	<0.5	<0.25	<1	<2	≥4/76
**248**	E	4	≥128	<4/2	<0.5	4	<0.5	4	<32	<0.5	<0.5	<1	<4	<1	<1	<0.5	<0.25	<1	<2	≥4/76
**330**	D	4	≥128	<4/2	<0.5	2	<0.5	4	<32	<0.5	<0.5	<1	<4	<1	<1	<0.5	<0.25	<1	<2	≥4/76
**321**	D	4	≥128	<4/2	<0.5	4	<0.5	4	<32	<0.5	<0.5	<1	<4	<1	<1	<0.5	<0.25	<1	<2	≥4/76
**57-1**	C	4	≥128	<4/2	<0.5	4	<0.5	4	<32	<0.5	<0.5	<1	<4	<1	<1	<0.5	<0.25	<1	<2	≥4/76
**55**	C	4	≥128	<4/2	<0.5	4	<0.5	4	<32	<0.5	<0.5	<1	<4	<1	<1	<0.5	<0.25	<1	4	≥4/76
**46-1**	C	4	≥128	<4/2	<0.5	4	<0.5	4	<32	<0.5	<0.5	<1	<4	<1	<1	<0.5	<0.25	2	<2	≥4/76
**246-1**	E	4	≥128	<4/2	<0.5	8	<0.5	4	<32	<0.5	<0.5	<1	<4	<1	2	<0.5	<0.25	<1	<2	≥4/76
**47-2**	C	4	64	<4/2	<0.5	8	<0.5	4	<32	<0.5	<0.5	<1	<4	<1	<1	<0.5	<0.25	<1	<2	≥4/76
**253-1**	E	4	≥128	<4/2	<0.5	8	<0.5	2	<32	<0.5	<0.5	<1	<4	<1	2	<0.5	<0.25	<1	4	≥4/76
**131**	A	4	64	<4/2	<0.5	4	<0.5	2	<32	<0.5	<0.5	<1	<4	<1	<1	<0.5	<0.25	<1	<2	<0.5/9.5
**126**	A	4	64	<4/2	<0.5	4	<0.5	2	<32	<0.5	<0.5	<1	<4	<1	<1	<0.5	<0.25	<1	<2	<0.5/9.5
**44-2**	C	4	8	<4/2	<0.5	2	<0.5	2	<32	<0.5	<0.5	<1	<4	<1	<1	<0.5	<0.25	<1	<2	<0.5/9.5
**323**	D	4	32	<4/2	<0.5	8	<0.5	2	<32	<0.5	<0.5	<1	<4	<1	<1	<0.5	<0.25	<1	<2	≥4/76
**331**	D	4	≥128	<4/2	<0.5	8	<0.5	4	<32	<0.5	<0.5	<1	<4	<1	2	<0.5	<0.25	<1	<2	≥4/76
**283-2**	B	4	≥128	<4/2	<0.5	16	1	<1	<32	<0.5	<0.5	<1	<4	<1	2	<0.5	<0.25	<1	<2	≥4/76
**53-1-1**	C	4	≥128	<4/2	≥32	≥64	≥32	2	<32	<0.5	<0.5	<1	<4	<1	<1	<0.5	<0.25	<1	≥32	≥4/76
**259-1**	E	4	≥128	<4/2	<0.5	8	<0.5	4	<32	<0.5	<0.5	<1	<4	<1	2	<0.5	<0.25	<1	<2	<0.5/9.5
**46-2**	C	4	64	<4/2	<0.5	4	<0.5	4	<32	<0.5	<0.5	<1	<4	<1	2	<0.5	<0.25	<1	<2	<0.5/9.5
**292-2**	B	4	≥128	4 / 4	<0.5	32	2	<1	<32	<0.5	<0.5	<1	<4	<1	2	<0.5	<0.25	<1	16	≥4/76
**41**	C	4	32	<4/2	<0.5	2	<0.5	<1	<32	<0.5	<0.5	<1	8	<1	<1	<0.5	<0.25	<1	<2	≥4/76
**59**	C	4	≥128	<4/2	<0.5	4	<0.5	2	64	<0.5	<0.5	<1	8	<1	<1	<0.5	<0.25	<1	<2	≥4/76
**53-2**	C	4	≥128	<4/2	<0.5	4	<0.5	4	<32	<0.5	<0.5	<1	8	<1	<1	<0.5	<0.25	<1	<2	≥4/76
**300-2**	B	4	<2	<4/2	<0.5	<1	<0.5	4	<32	<0.5	<0.5	<1	<4	<1	2	1	1	<1	16	<0.5/9.5
**101**	D	5	≥128	<4/2	<0.5	<1	<0.5	<1	<32	<0.5	<0.5	≥16	<4	<1	≥16	≥8	4≦	<1	4	≥4/76
**95**	D	5	32	<4/2	<0.5	<1	<0.5	<1	<32	<0.5	<0.5	≥16	<4	<1	≥16	≥8	4≦	<1	<2	≥4/76
**208**	D	5	64	<4/2	<0.5	<1	<0.5	<1	<32	<0.5	<0.5	≥16	<4	<1	≥16	≥8	4≦	<1	<2	≥4/76

**212**	D	5	≥128	<4/2	<0.5	<1	<0.5	<1	<32	<0.5	<0.5	≥16	<4	4	≥16	≥8	4≦	<1	<2	≥4/76
**210**	D	5	≥128	<4/2	<0.5	<1	<0.5	<1	<32	<0.5	<0.5	≥16	<4	8	≥16	≥8	4≦	<1	<2	≥4/76
**209**	D	5	64	<4/2	<0.5	<1	<0.5	<1	<32	<0.5	<0.5	≥16	<4	8	≥16	≥8	4≦	<1	<2	≥4/76
**333**	D	5	64	<4/2	<0.5	<1	<0.5	<1	<32	<0.5	<0.5	≥16	<4	8	≥16	≥8	4≦	<1	<2	≥4/76
**219**	D	5	64	<4/2	<0.5	<1	<0.5	<1	<32	<0.5	<0.5	≥16	<4	8	≥16	≥8	4≦	<1	<2	≥4/76
**211**	D	5	32	<4/2	<0.5	<1	<0.5	<1	<32	<0.5	<0.5	≥16	<4	8	≥16	≥8	4≦	<1	<2	≥4/76
**96-1**	D	5	≥128	<4/2	<0.5	<1	<0.5	<1	<32	<0.5	<0.5	≥16	<4	8	8	≥8	4≦	<1	<2	≥4/76
**98**	D	5	≥128	<4/2	<0.5	<1	<0.5	<1	<32	<0.5	<0.5	≥16	<4	4	8	≥8	4≦	<1	<2	≥4/76
**91**	D	5	64	<4/2	<0.5	<1	<0.5	<1	<32	<0.5	<0.5	≥16	<4	4	8	≥8	4≦	<1	<2	2/38
**99-1**	D	5	≥128	<4/2	<0.5	<1	<0.5	<1	<32	<0.5	<0.5	8	<4	2	2	4	4≦	<1	<2	<0.5/9.5
**401-1**	C	5	≥128	<4/2	<0.5	<1	<0.5	<1	<32	<0.5	<0.5	<1	<4	<1	<1	4	4≦	<1	<2	≥4/76
**94**	D	5	≥128	<4/2	<0.5	<1	<0.5	<1	<32	<0.5	<0.5	4	<4	8	8	2	2	<1	<2	≥4/76
**242**	E	5	16	<4/2	<0.5	4	<0.5	<1	<32	<0.5	<0.5	<1	8	<1	<1	≥8	4≦	<1	<2	≥4/76

Piperacillin (PIPC), Tazobactam/Piperacillin (TAZ/PIPC), Cefepime (CFPM), Ceftazidime (CAZ), Cefozopran (CZOP), Colistin (CL), Fosfomycin (FOM), Imipenem (IPM), Meropenem (MEPM), Gentamicin (GM), Amikacin (AMK), Tobramycin (TOB), Minocycline (MINO), Levofloxacin (LVFX), Ciprofloxacin (CPFX), Doripenem (DRPM), Aztreonam (AZT), and Trimethoprim/sulfamethoxazole (TMP/SMX).
