# Supplementary material for: Persistence of Colistin Resistance and mcr-1.1-Positive E. coli in Poultry Despite Colistin Ban in Japan
Source: Antibiotics (Basel). 2025 Apr 1;14(4):360. doi: 10.3390/antibiotics14040360 (PMC12024320; doi:10.3390/antibiotics14040360)
Supplement: Supplementary file 1 [file antibiotics-14-00360-s001.zip › Table-S2.pdf]

Supplementary Table S2. Whole genome sequence information of *E. coli* ST1485 strains.

Strain	BioProject	BioSample	DRA_Experiment	DRA_Run	Assembly
BroCaecum-17-2	PRJDB19974	SAMD00874694	DRX613973	DRR633647	BAAGHP010000001-BAAGHP010000128
BroCaecum-53-1-1	PRJDB19974	SAMD00874695	DRX613991	DRR633665	BAAGHQ010000001-BAAGHQ010000119
BroCaecum-55	PRJDB19974	SAMD00874696	DRX613992	DRR633666	AP039418-AP039422
BroCaecum-135	PRJDB19974	SAMD00874697	DRX613972	DRR633646	BAAGHR010000001-BAAGHR010000109
BroCaecum-258	PRJDB19974	SAMD00874698	DRX613979	DRR633653	BAAGHS010000001-BAAGHS010000100
BroCaecum-321	PRJDB19974	SAMD00874699	DRX613983	DRR633657	BAAGHT010000001-BAAGHT010000100
BroCaecum-323	PRJDB19974	SAMD00874700	DRX613984	DRR633658	BAAGHU010000001-BAAGHU010000103
